# GRAY HAIR AND PINK SLIPS: AN ANALYSIS OF TWITTER RESPONSES TO GENDERED AGEISM

**DOI:** 10.1093/geroni/igad104.2706

**Published:** 2023-12-21

**Authors:** Anne Barrett, Skyler Bastow, Rachael Dominguez, Melissa Frost, Cherish Michael, Hope Mimbs, Brianna Soulie

**Affiliations:** Florida State University, Tallahassee, Florida, United States; Florida State University, Tallahassee, Florida, United States; Florida State University, Tallahassee, Florida, United States; Florida State University, Tallahassee, Florida, United States; Florida State University, Tallahassee, Florida, United States; Florida State University, Tallahassee, Florida, United States; Florida State University, Tallahassee, Florida, United States

## Abstract

Popular, long-time Canadian broadcaster, Lisa LaFlamme, was fired in August 2022, with some observers suggesting that CTV National News did not renew her contract because she had “let her hair go gray” during the pandemic. Over the next several months, an international public outcry ensued on Twitter. Our study involved an analysis of over 400 of these tweets. Analyses revealed that over two-thirds of tweets indicated support for LaFlamme’s position, with nearly all the remaining tweets indicating neutral rather than negative positions. Frequent themes in the supportive tweets included assessments of her firing as undeserved and the actions of the news corporation as unjust. Calls to boycott the corporation also appeared. Other themes found in supportive tweets included ageism and sexism; however, references to sexism were somewhat more frequent than those to ageism. A theme appearing not only in supportive but also in neutral or oppositional tweets centered on beauty standards, with supportive tweets more likely to critique these standards’ sexist and ageist underpinnings. Our study extends the literature on gendered ageism by attending to collective responses to it. In contrast, prior studies have focused on individual-level experiences of and responses to gendered ageism. Our study also contributes to the hashtag activism literature, which has given little attention to efforts to dismantle age inequality.

